# Can salivary lactate be used as an anaerobic biomarker?

**DOI:** 10.7717/peerj.15274

**Published:** 2023-05-02

**Authors:** Pingping Yan, Chunli Qin, Zengyin Yan, Chang Chen, Fengjing Zhang

**Affiliations:** 1 College of Exercise Medicine, Chongqing Medical University, Chongqing, China; 2 Chongqing Institute of Sport Science, Chongqing, China; 3 School of Physical Education, Chongqing University of Posts and Telecommunications, Chongqing, China; 4 Institute of Life Sciences, Chongqing Medical University, Chongqing, China; 5 Stomatological Hospital of Chongqing Medical University, Chongqing, China; 6 Chongqing Key Laboratory of Oral Diseases and Biomedical Sciences, Chongqing, China; 7 Chongqing Municipal Key Laboratory of Oral Biomedical Engineering of Higher Education, Chongqing, China

**Keywords:** Lactate, Saliva, Biomarker, Anaerobic, LC-MS

## Abstract

**Background:**

Salivary lactate has been suggested as a non-invasive anaerobic biomarker in sports medicine for decades, yet has not been widely applied until now. This study aimed to explore possible issues related to its application and suggest directions for future method improvement.

**Methods:**

A liquid chromatography–mass spectrometry (LC-MS) method for the determination of salivary lactate was developed, validated and applied on saliva samples collected from a group of professional sprinters (*n* = 20). The samples were collected via chewing a cotton ball for one minute and centrifuging it afterwards. The evaluation included variation with mouth rinse times, consistency at different sampling times, change after treadmill or cycle ergometer trainings, and association with blood lactate. Sample sizes were calculated prior to the study. One-way analysis of variance (ANOVA), intra-class correlation coefficients (ICC) and relative standard deviation (RSD) were used to evaluate data variances. Pearson correlation was applied to show correlation between salivary and blood lactate. Effect sizes and power were calculated following ANOVA and correlation analyses.

**Results:**

The RSD of the LC-MS method was 19.70%. Salivary lactate concentration was affected by mouth rinse times before sampling (ANOVA *p* = 0.025, *η*^2^ = 0.40, 1 − *β* = 0.99, ICC = 0.23, mean RSD of four sampling = 55.30%), and stabilized after mouth rinsing for three times. The concentrations at resting state across three weeks were consistent at group level (ANOVA *p* = 0.57, *η*^2^ = 0.03, 1 − *β* = 0.20), but varied greatly individually (ICC = 0.22, mean RSD = 56.16%). Salivary lactate level significantly increased after treadmill and cycle ergometer trainings (ANOVA *p* = 0.0002, *η*^2^ = 0.46, 1 − *β* = 0.9999 and ANOVA *p* = 0.0019, *η*^2^ = 0.40, 1 − *β* = 0.9993, respectively), and displayed positive correlation with blood lactate concentration (*r* = 0.61, *p* = 0.0004, 1 − *β* = 0.9596). Significant difference between male and female participants was observed in none of the tests conducted in this study.

**Discussion:**

Salivary lactate was found to be a potential anaerobic biomarker. However, reproducible methods for sample collection and analysis, as well as more knowledge on the secretion mechanism and pattern of salivary lactate are required to make it a practical anaerobic biomarker.

## Introduction

Objective and timely evaluation of an athlete’s anaerobic capacity is of great significance in sports medicine (Noordhof, De Koning & Foster, 2010). Biomarkers are substances in biospecimen that objectively reflect the physiological and pathological processes within the body. Blood lactate, whose concentration increases with the load of anaerobic exercise, is currently regarded as the “gold standard” to evaluate an athlete’s anaerobic capacity (Billat, 1996). The measurement of blood lactate has been simplified with portable lactate analyzers (https://www.ekfdiagnostics.com/lactate-scout.html). However, blood sampling test is an invasive test, which brings pain, stress, and risk of infection (Segura et al., 1996). Therefore, a non-invasive test may be welcome if it has equivalent accuracy in evaluating an athlete’s anaerobic capacity.

Saliva is a type of non-invasive biological sample that can be easily collected. Positive correlation between salivary lactate and blood lactate (Mendez, Franklin & Kollias, 1976; Ohkuwa et al., 1995; Santos et al., 2006; Bocanegra et al., 2012; Tekus et al., 2012), as well as elevated salivary lactate level after intensive physical exercises (Bardon, Ceder & Kollberg, 1983; Ohkuwa et al., 1995; Santos et al., 2006; Tekus et al., 2012; Volodchenko et al., 2019) were observed in a number of studies. Segura et al. (1996) concluded that salivary lactate can be used as an alternative to blood lactate after a series of validation work. In the following year, Calvo et al. (1997) further introduced the concept of salivary lactate threshold, and displayed high correlation between blood lactate threshold and salivary lactate threshold (*r* = 0.93, *p* < 0.001). Despite these promising data generated decades ago, salivary lactate is still not widely applied in sports medicine nowadays. In this study, we aimed to explore what might have prevented salivary lactate from being used in practice.

Specifically, factors that potentially affect the analysis of salivary lactate, including analytical method, mouth rinses before sampling, and data consistency at different sampling days were evaluated. Elevation of salivary lactate during and after two types of short-time high-intensity trainings, and its correlation with blood lactate were also verified.

A customized liquid chromatography–mass spectrometry (LC–MS) method was developed and validated for the analysis of salivary lactate. This method was chosen for its high throughput. In fact, we also simultaneously measured 23 other potential salivary biomarkers of physical activities, including creatinine, uric acid, urea, cortisol, testosterone, choline, 5-hydroxyindole acetic acid, homovanillic acid and 15 essential amino acids together with lactate, with the hope of discovering new salivary biomarkers in sports. However, these 23 compounds were either undetected or unchanged after the exercise training, and hence the data were not shown in this report.

## Materials & Methods

### Samples

The participants were 20 professional sprinters, including eleven females and nine males. Six of them were 100-meter runners, three were 200-meter runners and eleven were 400-meter runners. They had been engaged in training for three years or longer at a training center where the study was conducted. Verbal informed consent from the athletes and their coaches were obtained prior to sampling. All the participants fulfilled the following criteria: (1) they were in good health, without sport injuries or bleeding wounds in the mouths, nor underwent dental surgery in the last three months; (2) they did not smoke, drink alcohol, or participate in strenuous physical activities 24 h prior to the study; and (3) they did not eat food or chew gum two hours prior to sampling. The study was conformed to the Declaration of Helsinki and approved by the ethics committee of the Stomatological Hospital of Chongqing Medical University, with the approval number of 2021(061).

To obtain a saliva sample, a medical cotton ball weighing approximately 0.3 g was chewed for one minute, and spitted into a 10 ml centrifuge tube. The tube was immediately sealed and temporarily stored in an ice box for no more than 2 h before transported to the laboratory and stored at −20 °C.

The evaluation was consisted of five parts: (1) validation of method accuracy and precision, (2) the influence of mouth rinses, (3) consistency at resting state, (4) variation during training, and (5) correlation with blood lactate. Accordingly, the participants were asked to provide saliva samples as follows: (1) at resting state, before and after one, two and three times of mouth rinses with 10 ml of drinking water (four females and six males); (2) at resting state, before breakfast (approximately 7 am) in Monday mornings of three consecutive weeks and after three times of mouth rinses (11 females and nine males); and (3) five minutes before, immediately after and five minutes after acute high-intensity treadmill training with increasing load or cycle ergometer trainings (eight females and eight males) after three times of mouth rinses.

As many professional sprinters in the training center as possible were recruited into this study. Sample sizes required were calculated based on the effect sizes of previous similar studies (Bardon, Ceder & Kollberg, 1983; Ohkuwa et al., 1995; Segura et al., 1996; Calvo et al., 1997; Bocanegra et al., 2012; Tekus et al., 2012), the significant level of 0.05 and the power of 0.8, which were 6 (95% CI: 0–13) for paired group comparisons and 11 (95% CI: 3–20) for Pearson correlation analysis, respectively. Hence, the planned sample sizes (10, 20 and 16 for paired group comparisons and 32 for Pearson correlation analysis of lactate concentrations, respectively) were larger than the required sample sizes (6 for paired group comparisons and 11 for Pearson correlation analysis of lactate concentrations, respectively).

The treadmill and cycle ergometer trainings started at approximately 9 am on two Thursday mornings with a one-month interval. The participants provided their information including sex, age, height and body weight, and sat quietly for 20 min prior to the trainings. The acute high-intensity treadmill training with increasing load was conducted with a Metalyzer 3B (Cortex, Leipzig, Germany) spiroergometry system and a RUN 7410 treadmill (RUNNER, Modena, Italy) preheated for 30 min. The training included five-minute warm-up at 8 km/h and 0° grade, 8 min load-increasing stage with increasing speed and slope every minute based on the participants’ individual capacities, and three-minute recovery stage at 5–7 km/h, 0° grade. The participants were equipped with heart rate monitors and accompanied by a team doctor. They were encouraged to complete the training but allowed to stop anytime when exhausted. The exhaustion criteria were set as the heart rate remaining at no less than 180 beats/min for two minutes, and the rate of perceived exertion (RPE) scale (Borg, 1982) reaching 18–20. The cycle ergometer training was similar to the Wingate test, which was completed on a Monark 874E weight cycle ergometer. Each participant was asked to ride on the cycle ergometer for 3 × 30 s with 3 min intervals at the load of 0.075 kg/kg body weight. Blood lactate was measured before and immediately after the cycle ergometer training using a Lactate Scout Lactate analyzer (EKF Diagnostics, Cardiff, UK), and transformed from mmol/L to µg/ml. Since the actual sample size of blood lactate was more than double of the required sample size, no blood lactate test was conducted during the treadmill training to reduce the participants’ stress.

### Instruments and procedures

Prior to analysis, the cotton ball soaked with saliva was defrosted at room temperature and transferred into a 4 ml Eppendorf tube with a small hole drilled at the bottom. The 4 ml tube was then put into a clean 10 ml Eppendorf tube whose cap was cut off. The inner diameter of the 10 ml Eppendorf tube was just between the outer diameters of the body and edge of the 4 ml Eppendorf tube, enabling the 4 ml tube to be stuck at the top of the 10 ml tube. After that, the 10 ml tube was centrifuged with an Eppendorf Centrifuge 5920 R (Hamburg, Germany) at 4 °C and 3,000 rpm for 3 min, so that the saliva in the cotton ball was collected in the 10 ml Eppendorf tube. 100 µl of the saliva sample was transfer into a 1.5 ml Eppendorf tube, and vortex-mixed with 20 µl of 1 µg/ml internal standard 2-Cl-phenylalanine. Then, 1 ml of acetonitrile was added and the mixture vortexed again. After that, the 1.5 ml centrifuge tube was centrifuged with an Eppendorf Centrifuge 5427 R (Hamburg, Germany) at 4 °C and 10,000 rpm for 10 min. The supernatant was transferred to a new 1.5 ml Eppendorf tube, dried with a Labconco CentriVap vacuum dryer (MO, USA) at 40 °C, and then reconstituted in 100 µl of 5% methanol. The solution was centrifuged again at 4 °C, 10,000 rpm for 5 min and 80 µl of the supernatant was transfer to an LC-MS vial for injection. Calibration samples were prepared in the same way (starting from internal standard addition) as the saliva samples. A pool sample was prepared by mixing 100 µl aliquots of all the test samples and used in method validation.

The LC-MS analysis was performed using an Agilent 1290-6495C Ultra High Performance Liquid Chromatography-Triple Quadrupole Tandem Mass Spectrometry (CA, USA). LC separation was performed using a Waters HSS T3 (100 ×2.1 mm, 1.7 µm) column with a Waters ACQUITY UPLC HSS T3 VanGuard (2.1 × 5 mm, 1.8 µm) pre-column. Mobile phase A was aqueous solution of 0.1% formic acid, and mobile phase B was acetonitrile. The gradients were: 0–0.5 min: 5% B, 0.5–1 min: 5–50% B, 1–7.5 min: 50% B, 7.5–8 min: 50–5% B, 8–10 min: 5% B. The flow rate was 0.3 ml/min, the injection volume was 2 µl, and the column temperature was 40 °C. The ion source parameters for the mass spectrometry were: Gas Temp: 200 °C, Gas Flow: 14 L/min, Nebulizer: 35 psi, Sheath Gas Temp: 300 °C, Sheath Gas flow: 11 L/min, Capillary: −3000 V, Nozzle Voltage: 500 V, iFunnel H: −150 V, L: −60 V. Multiple reaction monitoring (MRM) mode was used to analyze lactate and the internal standard 2-Cl-phenylalanine. Q1(m/z), Q3(m/z) and CV(eV) were set as 89.0, 43.2 and 13, respectively, for lactate; and 198.0, 181.0 and 9, respectively, for 2-Cl-phenylalanine. Lactate standard was purchased from Bidepharm (Shanghai, China), and 2-Cl-phenylalanine was from MedChemExpress (Shanghai, China).

The method was validated for accuracy and precision. Accuracy was determined by adding 10 µl/ml of lactate into a pool sample and calculating the recovery, which was the ratio of measured concentration increase to the theoretical concentration increase (10 µl/ml). Precision was determined by analyzing seven 100 µl sub-aliquots of the pool sample twice, and calculating the relative standard deviation (RSD) of the fourteen measured concentrations.

Each set of data is expressed as mean ± standard deviation (SD). Paired one-way analysis of variance (ANOVA) and Tukey’ post-hoc multiple comparisons test were used to compare data among different time points of the same group of participants, and multiplicity adjusted *P* value for each comparison with family-wise significance and confidence level set at 0.05 was reported. Intra-class correlation coefficients (ICC) and RSD of multiple sampling times were used to evaluate data variances of each participant. Pearson correlation coefficient (r) was calculated for salivary lactate and blood lactate of the same participants at the same time points. Effect sizes and power were calculated following ANOVA and correlation analyses. ANOVA, RSD and Pearson correlation were calculated using GraphPad Prism 8 for mac (GraphPad Software, Boston, MA, USA), while ICC was calculated with IBM SPSS Statistics software, version 20.0.0 (IBM Corporation, Armonk, NY, USA). Effect sizes of ANOVA (*η*^2^) were calculated from ANOVA F values, using the equation *η*^2^ = F × (k−1) / [F × (k−1) + (n−k)] (Cohen, 1988), while those of Tukey’s post-hoc tests (Cohen’s d) and t-tests were calculated with R for Mac OS X GUI 1.70 (R Core Team, 2020) and package “esc” (Lüdecke, 2019). Power (1–*β*) and sample sizes of all analyses were calculated with R software and package “pwr” (Champely et al., 2020).

## Results

**Figure 1:**
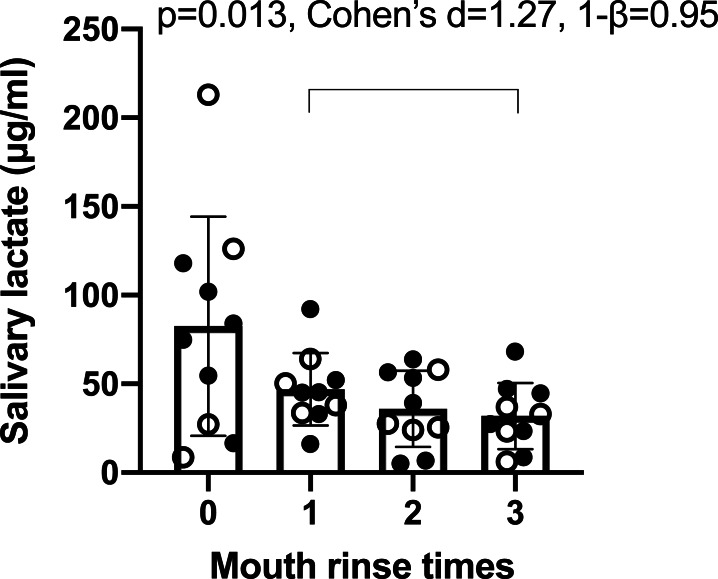
Concentration of salivary lactate after zero, one, two and three times of mouth rinse. Data are mean ± SD, *n* = 11. Solid dots represent male participants, while hollow dots represent female participants. Post-hoc analysis showed significant difference between one and three times of mouth rinse. The *p* value, effect size (Cohen’s d) and power (1–*β*) are listed on the figure.

It was calculated that the method accuracy was 95.9% and the RSD was 19.7%. Using this method, it was found that the times of mouth rinses (0, 1, 2, 3) significant affected salivary lactate levels (Fig. 1). ANOVA p was 0.025, effect size (*η*^2^) was 0.40, power (1–*β*) was 0.99, ICC was 0.23, and mean RSD of four sampling was 55.30%. The average concentration decrease with the increasing times of mouth rinses. After three times of mouth rinses, the salivary lactate concentration was significantly lower than that after one rinse, so three times of mouth rinses prior to sampling was applied in the following evaluations.

**Figure 2:**
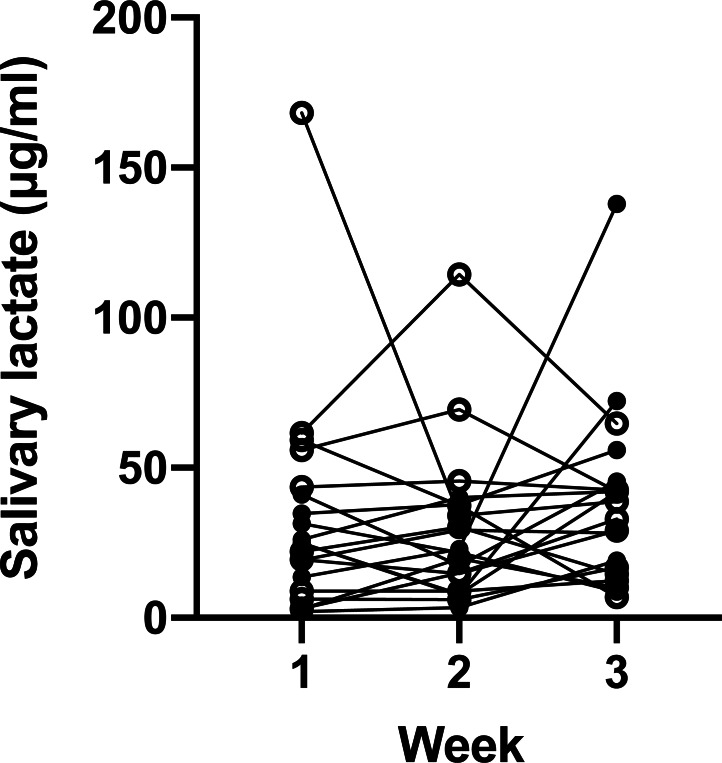
Salivary lactate levels of athletes at resting state on three consecutive Monday mornings. Data are mean ± SD, *n* = 20. Solid dots represent male participants, while hollow dots represent female participants. ANOVA *p* = 0.57.

We then investigated whether the salivary lactate concentration was consistent at different sampling days *via* analyzing samples collected once a week (Fig. 2). ANOVA showed that the average concentration of the 20 participants did not differ from week to week (*p* = 0.57, effect size (*η*^2^) = 0.03, power (1–*β*) = 0.20). However, marked individual variance was observed, which was shown by an ICC of 0.22 and a mean RSD of 56.16%.

**Figure 3:**
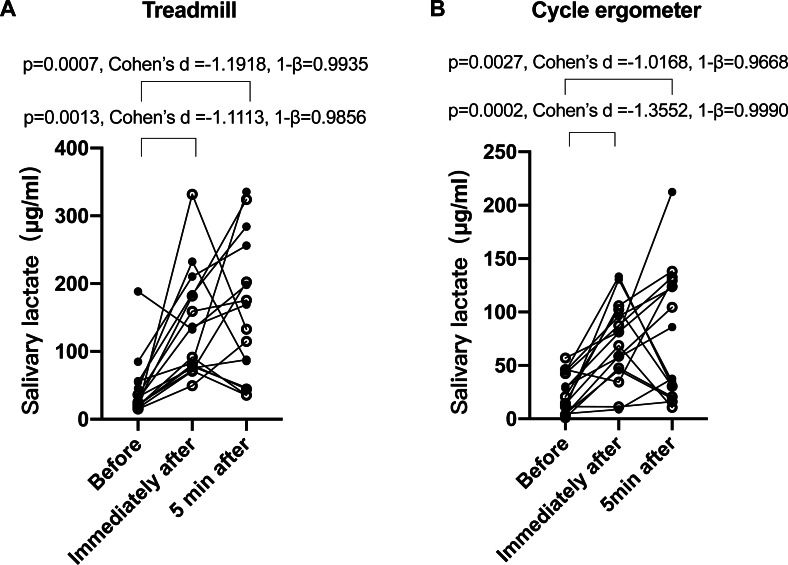
Salivary lactate concentrations at different sampling points of treadmill and bicycle ergometer trainings. (A) Treadmill; (B) bicycle ergometer. Data are mean ± SD, *n* = 16. Solid dots represent male participants, while hollow dots represent female participants. *P* values, effect size (Cohen’s d) and power (1–*β*) of Tukey’s *post-hoc* tests are listed on the figures.

Next, we verified whether salivary lactate increased after training. Despite large inter-individual variances, it was clear that the salivary lactate levels significantly increased after the treadmill (Fig. 3A) and cycle ergometer (Fig. 3B) trainings. ANOVA *p* value was 0.0002, effect size (*η*^2^) was 0.46, and power (1–*β*) was 0.9999 for treadmill trainings; while ANOVA *p* value was 0.0019, effect size (*η*^2^) was 0.40, and power (1–*β*) was 0.9993 for cycle ergometer trainings. There was no significant difference between immediately and 5 min after training.

**Figure 4:**
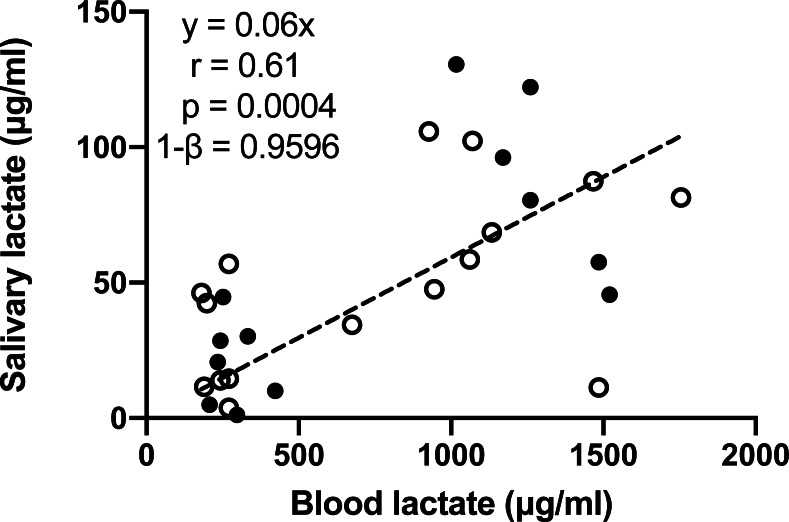
Pearson correlation analysis of salivary and blood lactate in samples collected before and immediately after the bicycle ergometer training. Data are mean ± SD, *n* = 29. Solid dots represent male participants, while hollow dots represent female participants.

One participant missed the blood lactate test, and another participant missed one time point of the blood lactate test on the day of cycle ergometer training. Consequently, 14 out of the 16 participants underwent the blood lactate before and immediately after the cycle ergometer training, and one out of the 16 participants only underwent blood lactate test at one time point, which provided a total of 14 × 2 + 1 = 29 pairs of blood-salivary lactate data. The 29 pairs of salivary-blood lactate data showed significant and positive correlation (Fig. 4). The 95% confidence interval of correlation coefficient was 0.32–0.80. Significant difference between male and female participants was observed in none of the tests conducted in this study.

## Discussion

In this study, we evaluated whether salivary lactate can be used as an anaerobic biomarker. Significant increase of salivary lactate during and after two forms of short-time high-intensity exercises (Fig. 3), as well as positive correlation between salivary and blood lactate (Fig. 4), were observed. These observations were consistent with previous studies (Mendez, Franklin & Kollias, 1976; Bardon, Ceder & Kollberg, 1983; Ohkuwa et al., 1995; Santos et al., 2006; Bocanegra et al., 2012; Tekus et al., 2012; Volodchenko et al., 2019). However, the correlation was overall moderate, which did not support the replacement of blood lactate with salivary lactate. Based on the displayed data, this may be due to three reasons.

Firstly, the production of saliva lactate was influenced by multiple factors. Even with the same sampling procedures, at the same time of day and day of week, saliva samples were found to vary, with the RSD as high as 56.16% (Fig. 2). It should be noted that this RSD may be overestimated, because the samples were obtained by the participants without supervision, and hence the sampling instructions might not be strictly followed. Chicharro et al. (1998) summarized that the saliva composition is regulated by nervous system, salivary flow rate, stimuli and other factors such as age, circadian rhythm, circannual rhythms and reflex. A recent study suggested that body fat, body water content and skeletal muscle mass index are associated with salivary lactate levels after exercise (Okano et al., 2022). Each of these factors may vary among individuals and change over time. Moreover, microbes in the oral cavity such as *Lactobacillus* produce lactate (Selle & Klaenhammer, 2013), which can be disolved in saliva.

Secondly, saliva collection methods may affect lactate concentration. Saliva is secreted at the rate of approximately 0.5 ml/min by three major pairs of salivary glands and a number of minor mucous glands, and the flow rate may increase with stimuli (Dawes, 1974). Thus, saliva samples can be obtained either in an unstimulated way, such as passively drool (Kawanishi et al., 2019), or a stimulated way such as that in this study. Unstimulated methods are reported to produce more consistent data but take longer to accumulate the required volume, and stimulated methods take less time but generate less consistent data (Kawanishi et al., 2019). We chose stimulated method in this study because the participants may have dry mouths after the trainings, so unstimulated methods may take too long. The RSD of 55.30% and ICC of 0.23% of salivary lactate were observed in the saliva samples collected after different mouth rinse times (Fig. 1). This indicated that sampling method and preparation prior to sampling had significant impact on salivary lactate. Our data also suggested that three times of mouth rinses prior to sample collection was able to stabilize salivary lactate. Previous studies applied different strategies for mouth cleaning prior to sampling, such as no mouth rinse (Hough et al., 2013), one mouth rinse (Liu et al., 2017) or tooth brushing (Volodchenko et al., 2019), which indicated that a standardized sampling protocol may be needed for future studies.

Finally, the precision of the analytical method was not satisfactory. In this study, an LC-MS method with an RSD of 19.70% was developed to analyze salivary lactate. In contrast, enzymatic methods for salivary lactate measurements normally produce an RSD of approximately 2% or less (Segura et al., 1996; Artisan Technology Group, 2000). This may be due to the saliva samples having to be prepared for LC-MS analysis, and the process included multiple steps such as pipetting, centrifugation, evaporation and reconstitution. Besides, no deuterated internal standard of lactate was applied in this method. All of these factors may have contributed to the relatively large RSD of the LC-MS method. In the future, simplified sample preparation procedures and application of a deuterated internal standard are recommended in the LC-MS analysis of salivary lactate.

## Conclusions

To summarize, salivary lactate is a promising biomarker for the evaluation of anaerobic capacities, but reliable sampling and analytical methods, together with comprehensive knowledge on the mechanism and pattern of salivary lactate secretion, are crucial elements to make it practical.

## Additional Information and Declarations

### Data Availability

The following information was supplied regarding data availability: The raw data is available at Zenodo: Fengjing Zhang. (2022). Raw data for “Can salivary lactate be used as an anaerobic biomarker”. https://doi.org/10.5281/zenodo.7592880.
